# Calorie-restricted diet mitigates weight gain and metabolic abnormalities in obese women with schizophrenia: a randomized controlled trial

**DOI:** 10.3389/fnut.2023.1038070

**Published:** 2023-05-05

**Authors:** Lei Zhang, Mingwen Zhu, Xiangqun Liu, Zhijun Zhao, Ping Han, Luxian Lv, Chun Yang, Yong Han

**Affiliations:** ^1^Department of Clinical Nutrition, The Second Affiliated Hospital of Xinxiang Medical University, Xinxiang, Henan, China; ^2^Department of Nutrition and Food Hygiene, School of Public Health, Zhengzhou University, Zhengzhou, Henan, China; ^3^Department of Psychiatry, The Second Affiliated Hospital of Xinxiang Medical University, Xinxiang, Henan, China; ^4^Henan Key Lab of Biological Psychiatry of Xinxiang Medical University, Xinxiang, Henan, China; ^5^Department of Nutrition and Food Hygiene, School of Public Health, Capital Medical University, Beijing, China

**Keywords:** calorie-restricted diet, obesity, women, schizophrenia, randomized controlled trial

## Abstract

**Background:**

Obesity is a prevalent health problem in patients with schizophrenia, and calorie restriction diet (CRD) achieved effective weight loss and metabolic improvement; however, these have not been rigorously evaluated in obese patients with schizophrenia.

**Objective:**

To measure the effects of CRD on weight loss and metabolic status in hospitalized obese women with schizophrenia during a 4-week period.

**Methods:**

Participants were randomly assigned to two groups in a 1:1 ratio. The intervention group (*n* = 47) was asked to follow a CRD and the control group (*n* = 48) a normal diet for 4 weeks. Outcomes of body weight, body composition, as well as metabolic parameters were measured at baseline and following the intervention period.

**Results:**

Forty-five participants completed the 4-week research in both the intervention and control groups. Compared to the normal diet, adherence to the CRD significantly decreased body weight (2.38 ± 1.30 kg), body mass index (0.94 ± 0.52 kg/m^2^), waist circumference (4.34 ± 2.75 cm), hip circumference (3.37 ± 2.36 cm), mid-upper circumferences, triceps skin-fold thickness, fat mass and free fat mass with large effect sizes (*p* = <0.001, *ηp*^2^ range between 0.145 and 0.571), as well as total cholesterol (0.69 ± 0.70 mmol/L) with a medium effect size (*p* = 0.028, *ηp*^2^ = 0.054). There were no differences between the CRD and control groups in terms of pre-post changes in triglycerides, high- and low-density lipoprotein-cholesterols, as well as systolic and diastolic blood pressures (*p* > 0.05).

**Conclusion:**

CRD is preventative of weight gain, but not apparent in intervention for metabolic status in hospitalized obese women with schizophrenia.

**Clinical trial registration**: http://www.chictr.org.cn, ChiCTR-INR-16009185.

## 1. Introduction

Schizophrenia is a common severe mental illness with a global prevalence of about 1%, among the world’s top ten causes of long-term disability (1). A fair amount of patients suffer from psychosis, apathy, and withdrawal, and cognitive impairment (2). What’s worse, 15–72% of patients experience sustained weight gain after treatment, especially in the first 12 weeks (3). The prevalence of obesity is twice as common in people with schizophrenia as in the general population (4), which is linked to many adverse consequences such as cardiovascular diseases (5), higher mortality (6), reduced quality of life (7), and poor compliance (8).

Obesity is a chronic metabolic condition that develops with the excessive accumulation of adipose tissue in the body (9). The cause of the increase in obesity for patients with schizophrenia is multifactorial, including poor diet (10, 11) and physical inactivity due to mental symptoms (such as laziness, passivity, etc.) and antipsychotics (12, 13) and genetic predisposition (14, 15). Antipsychotics are drugs that affect the activity of many hormones and neuromodulators that are well expressed in the hypothalamus, pancreas, liver, adipose tissue and skeletal muscle and regulate glucose and lipid homeostasis throughout the body, making it highly susceptible to metabolic side effects (16). Moreover, many studies have found that schizophrenia and metabolic abnormalities share familial risk factors or a common genetic predisposition, which may lead to schizophrenia being more prone to metabolic abnormalities than the general population (14, 15, 17). Sex is also believed to play a role in obesity among patients with schizophrenia, with a higher obesity rate among female patients (18). Currently, lifestyle intervention such as physical exercise and dietary regimens based on calorie restriction is known as the preferred treatment options of obesity (19). A number of pieces of evidence suggested that diet intervention, such as dietary approaches to stop hypertension (DASH) (20), very-low-calorie ketogenic diets (VLCKD) (21), low-fat vegan diet (LFVD) (22), very-low-calorie diet (VLCD) (23), and calorie-restricted diet (CRD) (24–27), safely achieve substantial weight loss and improvement of metabolic parameters in common population with obesity. The efficacy of CRD intervention has been confirmed in a variety of population (25), including health obesity subjects (28), obese patients with and without type-2 diabetes (29), obese patients with psoriasis (30), obese postmenopausal women with and without the metabolic syndrome (31). However, studies related to calorie-restricted diets in obese patients with schizophrenia are scarce. From now on, only one study of descriptive correlational design, utilizing chart review and a convenience sample of 100 participants, was used to evaluate the effect of CRD on weight change in short-term acute care psychiatric patients receiving atypical antipsychotic medication (32). Apparently, the results were lacking in persuasiveness due to flaws in the study design.

This study intends to use a randomized controlled trial to evaluate the effect of CRD on the body weight, body composition, and metabolic status of obese women with schizophrenia. We hypothesized that CRD is beneficial for body weight control and improving metabolic status in obese women with schizophrenia, comparing with general diet, thus offering a successful dietary prescription for use in clinical practice.

## 2. Methods

### 2.1. Study design and eligibility

This randomized clinical trial using a single-center, open parallel design was conducted between September 2016 and October 2017 in the Second Affiliated Hospital of Xinxiang Medical University (also known as Henan Mental Hospital). Female inpatients with schizophrenia aged 18 to 65 years with a body mass index (BMI) over 28 kg/m^2^ were enrolled. The Structured Clinical Interview for DSM-IV Axis I Disorders (SCID-I) patient version was used for the diagnosis of schizophrenia by two experienced psychiatric physicians. Exclusion criteria included: clinically significant body weight change (≥5 %) or dieting attempts in the prior 30 days; eating disorders such as anorexia or binge eating; diabetes, hypertension, dyslipidemia; need special diets due to physical diseases. To eliminate the possible impact of changes of drug regimens, diets, and lifestyles on metabolic indices after hospitalization, a 2-week run-in period was set before carrying out the study. Participants who met inclusion criteria 2 weeks after admission were randomized assigned to consume the CRD or normal diet (ND) for 4 weeks of intervention period (average length of stay 50 days for patients in the hospital). This study was a randomized controlled trial, and participants were randomly assigned to either CRD group or ND group using computer-generated random number allocation. Blinding of patients and study personnel was not possible given the nature of the study.

The trial adhered to the ethical guidelines of the Declaration of Helsinki and the Consolidated Standards of Reporting Trials (CONSORT) reporting guideline (33, 34). All subjects and their families in both groups voluntarily participated in this study with informed consent. This study was approved by the human ethics committees of the Second Affiliated Hospital of Xinxiang Medical University with the Approval number 2016023 and was registered at the Chinese Clinical Trials Registry^1^ with the registration number ChiCTR-INR-16009185 (data: September 10, 2016).

### 2.2. Intervention

After determining the group of intervention subjects, the clinical dietitian determines the energy intake of the subjects based on their past dietary history and anthropometric indicators. The energy distribution of breakfast, lunch, and dinner is roughly 30, 40, and 30%. Target calorie requirement of each patient was estimated based on resting energy expenditure (using Harris-Benedict equation) and physical activity level (35). The diet recipes are varied and do not repeat during the week, and the recipes are adjusted weekly according to the seasonal characteristics to choose fresh ingredients in season. Try to cook in healthy ways such as steaming, boiling, braising, boiling, and quick stir-frying.

The clinical dietitian conducted a nutritional check-up prior to the dietary intervention in order to know the patient’s eating habits, diet structure, and dietary contraindications (e.g., food allergies, ethnic habits, etc.). During the intervention period, the dietitian conducted weekly nutritional check-ups and kept an eye on the patients’ diet, asked them to avoid high-calorie snacks (such as nuts, instant noodle, alcohol, etc.) and allowed to consume fruits, tomato, cucumber, and plain milk in moderation. Since the food that patients obtained outside of their three meals is purchased through the commissary within the wards, the kinds and amounts of food they buy can be controlled within reasonable limits. Subjects’ charge nurses were responsible for monitoring their daily dietary compliance.

*CRD:* The dietary energy of CRD is reduced by about 500 kcal per day based on the target energy intake. The energy supply ratio of carbohydrates is 50–60%, fat is 20–30%, protein is 15–20%.

*ND:* Normal diet for control group followed a balanced diet with three macronutrients in appropriate proportions, with 55–65% carbohydrate, 20–30% fat, and 10–15% protein.

*Health education:* Individual nutrition counseling and dietary guidance for 30–60 min was offered to each participant of both groups within the first week of enrollment. In addition, weekly collective health lectures were held for 30–40 min each time, with topics including how to control weight, how to calculate and evaluate body mass index, types of food and their nutritional value, reasonable diet, scientific exercise, and the relationship between obesity and chronic diseases.

### 2.3. Outcomes measurements

Outcomes were measured at baseline and at week 4 from both groups. Body weight and composition were measured by body fat analyser (IOI 353, Danilsmc Co.,Ltd). Blood samples were drawn from the anterior elbow vein after one overnight fast and tested in 2 h by the hospital laboratory using standard techniques. Fasting blood glucose (FBG), total cholesterol (TC), low-density lipoprotein cholesterol (LDL), high-density lipoprotein cholesterol (HDL), and triglycerides were tested using Bechmann automatic biochemistry analyzer. The oxidation enzyme method was applied for FBG, TC and TG was measured using CHOD-PAP method and GPO-PAP method, respectively. HDL and LDL were tested using direct quantitation method (peroxidase scavenging and surfactant scavenging, respectively). Height (cm) measured using the Xiheng pointer height and weight scale (Wuxi, Jiangsu) to the nearest 0.1 cm without shoes, wearing lightweight clothes, barefoot and the head positioned in the Frankfurt horizontal plane. Blood pressure (BP) was measured by a standard digital sphygmomanometer (Omron HEM-7136, Omron Healthcare, Inc., Lake Forest, IL) with patients in a sitting position after a 5-min rest.

### 2.4. Sample size determination

The mean and standard deviation of BW changes in CRD group (−1.3, 2.0) and NC group (0.4, 2.4) obtained based on a pilot study (10 cases in each group), which were used for the calculation of the sample size of this study. Both group sample sizes of 37 achieve 90% power to reject the null hypothesis of equal BW change means and with a significance level of 0.05 using a two-sided two-sample unequal-variance t-test by PASS 15 software (NCSS LLC, Kaysville, UT, USA). Allowing for a dropout rate of 20%, we would need at least 47 patients in each group.

### 2.5. Statistics analysis

Mean along with SD and percentage were carried out for describing numeral variables and categorical variables. Distribution of data related to normality was evaluated using Shapiro Wilk test. The general characteristics between the two groups were compared using independent samples Student’s *t*-test or the Mann–Whitney U test (according to data normality) for numeral variables and Chi-square test or Fisher’s exact test for categorical variables. To identify intragroup differences (pre- and post- 4-week intervention), we applied paired samples t-tests. Additionally, a 95% confidence interval (CI) for the mean of the change from baseline (Δ = post-test - pre-test) was used to analyze significant changes in the variables. In addition, Cohen’s *d* was calculated as effect size (ES), which is a standardized measurement based on SD differences, used as a guide for substantive significance; while *d* = 0.2 was considered a small effect, *d* = 0.5 was a medium effect and *d* = 0.8 was a large effect. The effects of CRD on variables of body weight (BW), body component, FBG, blood lipid profile and BP were performed by analysis of covariance (ANCOVA) using pretest values as the covariate. Partial eta squared effect sizes (*ηp*^2^) were also reported on group as an indicator of effect size of ANCOVA. Suggested norms for *ηp*^2^: small = 0.01; medium = 0.06; large = 0.14. value of *p* lower than 0.05 was considered statistically significant. All statistical analyses were conducted using the SPSS version 18 (SPSS Inc., Chicago, IL, USA).

## 3. Results

### 3.1. Characteristics of the participants

**Figure 1:**
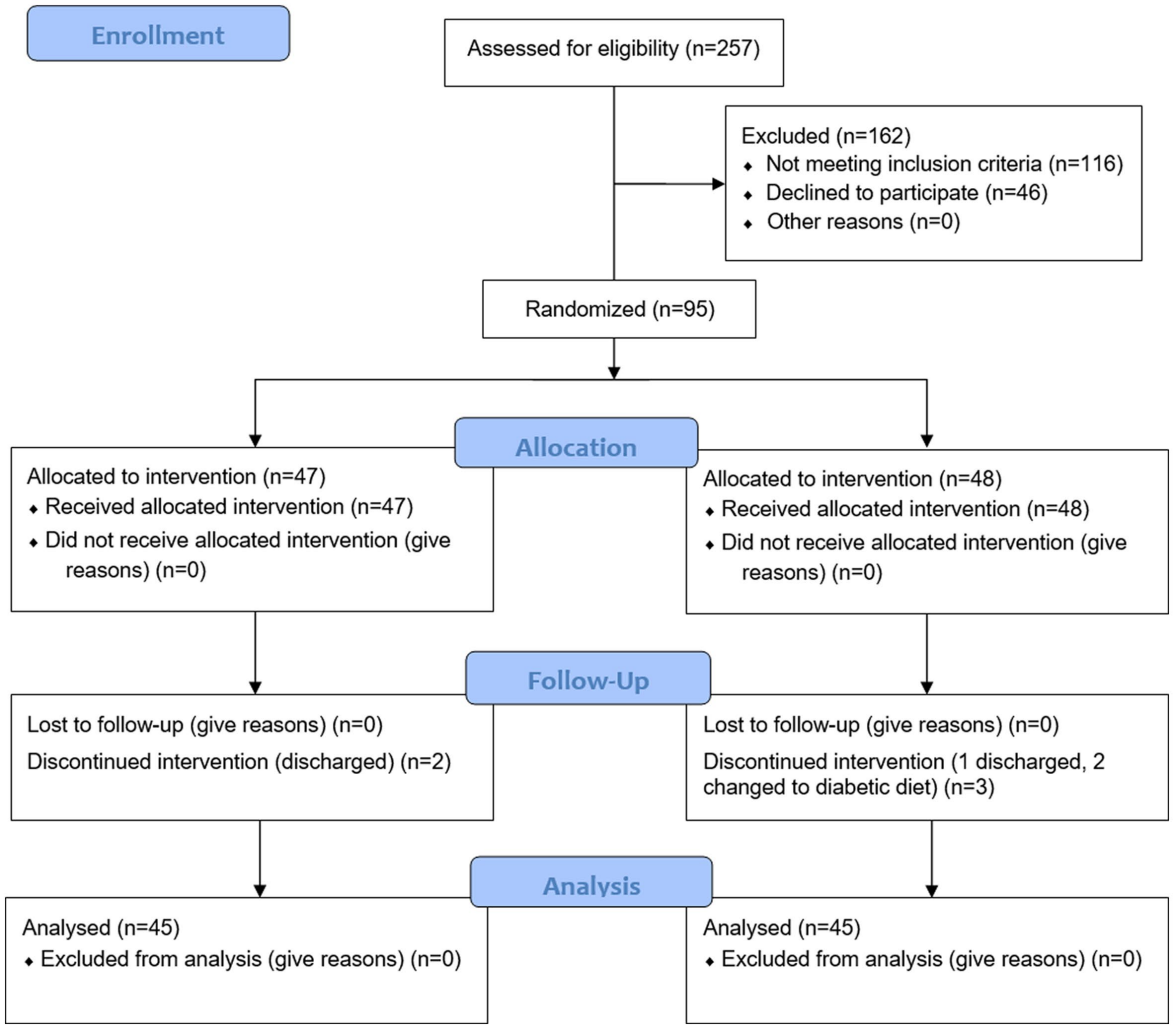
CONSORT flow diagram.

A total of 95 female inpatients with schizophrenia and obesity were included in this study, including 47 in the intervention group and 48 in the control group. Two subjects in the intervention group were discharged on the 12th and 17th day of the study. Three subjects in the control group failed to complete the intervention according to the trial protocol. Among them, 1 patient was discharged on the 7th day after the intervention started, and the other 2 patients developed hyperglycemia, whose diet were adjusted to diabetic diet. No harms or unintended effects were observed in either group by diet intervention throughout the trial. Ninety subjects were included in the analysis, with the intervention group and control group 45 cases, respectively. The following medications were used by the patients either singly or in combination: risperidone (*n* = 53), olanzapine (*n* = 29), levomepromazine (*n* = 28), and aripiprazole (*n* = 24). Figure 1 presents a CONSORT diagram.

The average age of the study subjects included in the final data analysis was 35.63 ± 10.79 years old, and the average BMI was 31.40 ± 2.44 kg/m^2^. Descriptive statistics with baseline characteristics are summarized by groups in Table 1, and there were no statistical significances in demography (age, education, occupation, marriage), metabolic variables (FBG, lipid profile, BP), and mental illness status between two groups. The seven-day dietary schedule of a subject in CRD group and a seven-day dietary schedule of the ND group were sampled randomly to analysis for representing the intake of calories, macronutrients, and cholesterol between the two groups. The results are shown in Supplementary Table 1.

**Table 1:** Baseline characteristics of participants.

	Intervention group (*n* = 45)	Control group (*n* = 45)	*t/χ* ^2^	Value of *p*
Demography				
*Age, years*	37.02 ± 10.56	34.24 ± 10.96	−1.220	0.224
*Education*			2.464	0.482
Elementary school or below	13	10		
Middle School	18	15		
Senior High School	7	13		
University or above	7	7		
*Occupation*				
Farmer	34	34	<0.001	1.000
Others	11	11		
*Marriage*			0.811	0.906
Unmarried	10	11		
Married	28	27		
Divorced	4	4		
Remarried	4	2		
Psychiatric history				
Family history			0.090	0.764
Yes	6	7		
No	39	38		
Disease duration, year	10.14 ± 6.98	9.36 ± 7.05	−0.530	0.595
Anthropometric indicators				
Height, cm	159.05 ± 5.58	159.91 ± 4.79	0.783	0.436
BMI, kg/m^2^	31.84 ± 2.82	30.95 ± 1.90	−1.754	0.083
BW, kg	80.63 ± 8.97	79.25 ± 7.24	−0.802	0.425
Metabolic indicators				
FBG, mmol/L	5.34 ± 0.89	5.04 ± 0.66	−1.827	0.071
TC, mmol/L	4.87 ± 0.69	4.64 ± 0.97	−1.247	0.216
TG, mmol/L	2.08 ± 1.97	1.59 ± 0.65	−1.576	0.119
SBP, mmHg	119.84 ± 12.94	117.16 ± 11.32	−1.049	0.297
DBP, mmHg	78.20 ± 8.27	76.02 ± 7.39	−1.317	0.191

BMI, body mass index; BW, body weight, FBG, fasting blood-glucose; TC, total cholesterol; TG, triglycerides; SBP, systolic blood pressure; DBP, diastolic blood pressure.

### 3.2. Body weight and composition

**Figure 2:**
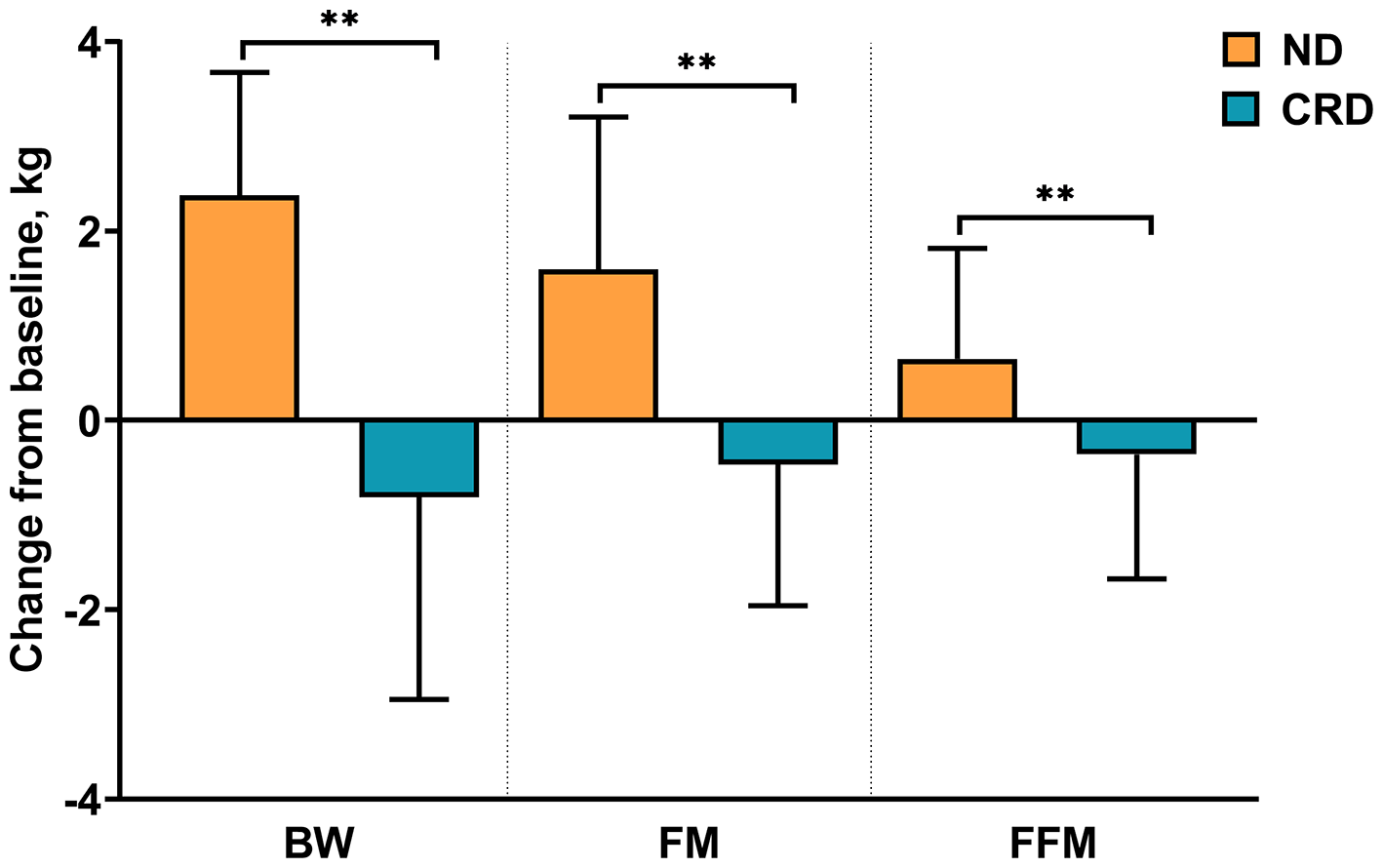
Changes from baseline in BW (body weight), FM (fat mass), and FFM (fat-free mass). ^**^ presents the changes between calorie restriction diet (CRD) group and normal diet (ND) group considered statistical significance (*p* < 0.001).

After 4 weeks of calorie-restricted diets, compared to baseline, BW, body mass index (BMI), waist circumference (WC), hip circumference (HC), mid-upper arm circumference (MUAC), triceps skinfold thickness (TST), fat mass (FM) and free-fat mass (FFM) showed a significant reduction in CRD group, among which the decrease of BW (−2.38 ± 1.30), BMI (−0.94 ± 0.52) and FM (−1.59 ± 1.61) showed a moderate effect size (*d* > 0.2) and WC (−4.34 ± 2.75), HC (−3.37 ± 2.36), MUAC (−1.26 ± 1.71) and TST (−3.80 ± 3.17) showed a large effect size (*d* > 0.5), while, only FFM (−0.65 ± 1.17) showed small effect size (*d* = −0.141). Regarding control group, except for the MUAC (0.16 ± 1.01, *p* = 0.300) and FFM (0.36 ± 1.31, *p* = 0.073), all the above indicators demonstrate an increased change with statistic difference after 4 weeks compared with the beginning. In particular, the skinfold showed moderate effect size (*d* = 0.216), but BW, BMI, WC, HC, FM showed small effect size. According to the results by group, significant differences were observed over group in all the above indicators (*p* < 0.001) after 4 weeks test, as well as with a large effect size for all (*ηp*^2^ > 0.12). In addition, the same statistical analysis was implemented in indicators of FBG, lipid profiles, and BP (see Table 2 and Figure 2 for details).

**Table 2:** Changes in body weight and composition during the study in the CRD intervention group vs. the ND group (mean ± SD).

Variables	Group	Baseline	End-of-trial	Change	*p* ^a^	Cohen’*d*	*p* ^b^	*ηp* ^2^
BW, kg	ND	79.25 ± 7.24	80.06 ± 7.35	0.8 ± 2.14	0.014	0.111	<0.001	0.452
	CRD	80.63 ± 8.97	78.25 ± 8.57	−2.38 ± 1.30	<0.001	−0.271		
BMI, kg/m^2^	ND	30.95 ± 1.90	31.28 ± 2.07	0.32 ± 0.87	0.017	0.161	<0.001	0.424
	CRD	31.84 ± 2.82	30.90 ± 2.64	−0.94 ± 0.52	<0.001	−0.345		
WC, cm	ND	106.10 ± 6.15	107.24 ± 5.84	1.14 ± 2.41	0.003	0.190	<0.001	0.571
	CRD	106.40 ± 6.51	102.06 ± 5.82	−4.34 ± 2.75	<0.001	−0.703		
HC, cm	ND	108.55 ± 5.99	109.56 ± 5.63	1.01 ± 2.22	0.004	0.174	<0.001	0.485
	CRD	110.06 ± 6.15	106.69 ± 5.89	−3.37 ± 2.36	<0.001	−0.56		
MUAC, cm	ND	31.81 ± 2.49	31.97 ± 2.65	0.16 ± 1.01	0.300	0.061	<0.001	0.197
	CRD	32.37 ± 2.29	31.12 ± 2.62	−1.26 ± 1.71	<0.001	−0.510		
TST, cm	ND	30.44 ± 5.75	31.64 ± 5.37	1.20 ± 2.28	0.001	0.216	<0.001	0.469
	CRD	32.10 ± 6.13	28.30 ± 5.30	−3.80 ± 3.17	<0.001	−0.662		
FM, kg	ND	29.83 ± 4.05	30.30 ± 3.92	0.47 ± 1.49	0.042	0.117	<0.001	0.304
	CRD	31.10 ± 4.61	29.51 ± 4.02	−1.59 ± 1.61	<0.001	−0.368		
FFM, kg	ND	44.87 ± 3.65	45.23 ± 3.80	0.36 ± 1.31	0.073	0.097	<0.001	0.145
	CRD	45.01 ± 4.63	44.36 ± 4.55	−0.65 ± 1.17	0.001	−0.141		

*ηp*^2^, partial eta squared effect size; BW, body weight; BMI, body mass index; WC, waist circumference; HC, hip circumference; MUAC, mid-upper arm circumference; TST, triceps skinfold thickness; FM, fat mass; FFM, free-fat mass.

### 3.3. FBG, blood lipid profiles, and BP

Regarding the FBG, there was a significant reduction in both groups compared to baseline, but the CRD show a large effect size (*d* = −0.730), while the CD group show a small effect (*d* = −0.382) (Table 3). With regard to blood lipid, the CRD group showed a significant reduction of both TC (−0.69 ± 0.70, *p* = <0.001) and low-density lipoprotein-cholesterol (LDL-C) (−0.40 ± 0.59, *p* = <0.001), along with moderate effect size (*d* > 0.5). Conversely, the ND group showed a significant increase of triglycerides (TG) (0.47 ± 0.93, *p* = 0.002), showing a moderate effect (*d* = 0.545). Adjustments for baseline values, compared with the control diet, the CRD dietary has resulted in significant reductions in TC (*p* = 0.028), but to a small size (*ηp*^2^ = 0.054).

**Table 3:** Changes in metabolic markers during the study in the CRD group vs. the ND group.

Variables	Group	Baseline	End-of-trial	Change	*p* ^a^	Cohen’*d*	*p* ^b^	*ηp* ^2^
FBG, mmol/L	ND	5.04 ± 0.66	4.77 ± 0.77	−0.27 ± 0.77	0.022	−0.382	0.361	0.010
	CRD	5.34 ± 0.89	4.78 ± 0.65	−0.57 ± 0.76	<0.001	−0.730		
TC, mmol/L	ND	4.64 ± 0.97	4.44 ± 0.94	−0.20 ± 1.06	0.202	−0.214	0.028	0.054
	CRD	4.87 ± 0.69	4.17 ± 0.69	−0.69 ± 0.70	<0.001	−1.001		
TG, mmol/L	ND	1.59 ± 0.65	2.06 ± 1.02	0.47 ± 0.93	0.002	0.545	0.076	0.036
	CRD	2.08 ± 1.97	1.83 ± 0.77	−0.25 ± 1.80	0.367	−0.164		
HDL-C, mmol/L	ND	1.19 ± 0.23	1.16 ± 0.29	−0.03 ± 0.28	0.475	−0.117	0.132	0.026
	CRD	1.16 ± 0.27	1.06 ± 0.28	−0.09 ± 0.32	0.056	−0.341		
LDL-C, mmol/L	ND	2.69 ± 0.61	2.61 ± 0.69	−0.08 ± 0.67	0.437	−0.121	0.100	0.031
	CRD	2.95 ± 0.74	2.55 ± 0.59	−0.40 ± 0.59	<0.001	−0.596		
SBP, mmHg	ND	117.16 ± 11.32	117.20 ± 12.32	0.04 ± 14.00	0.983	0.004	0.079	0.035
	CRD	119.84 ± 12.94	114.13 ± 9.90	−5.71 ± 12.45	0.004	−0.496		
DBP, mmHg	ND	76.02 ± 7.39	76.11 ± 8.17	0.09 ± 10.14	0.953	0.011	0.219	0.017
	CRD	78.20 ± 8.27	74.76 ± 7.48	−3.44 ± 8.46	0.009	−0.437		

FBG, fasting blood-glucose; TC, total cholesterol; TG, triglycerides; HDL-C, high density lipoprotein-cholesterol; LDL-C, low density lipoprotein-cholesterol; SBP, systolic blood pressure; DBP, diastolic blood pressure.

*p*^a^ indicated the *p*-value of inra-group comparison between baseline and end-of-trial; *p*^b^ indicated the *p*-value of inter-group comparison between changes of intervention group and control group.

### 3.4. Subgroups analysis

Subjects were split into three age subgroups, young (20 ≤ age < 30), middle-aged (30 ≤ age < 45) and elderly subgroup (45 ≤ age ≤ 60), and the foregoing analysis was conducted in each group (Supplementary Tables 2–7). Except for the middle-aged subgroup where the changes between the two groups in MUAC and FFM were not found, changes in all indicators of body weight and composition indicators were statistically different in all subgroups. Moreover, the statistical differences of TC changes, shown based on all subjects, were only found in the young subgroup, and both SBP (*p* = 0.011, *ηp*^2^ = 0.210) and DBP (*p* = 0.014, *ηp*^2^ = 0.196) in the middle-aged subgroup decreased significantly after the CRD intervention compared to ND.

## 4. Discussion

Our study aimed to evaluate the effect of CRD on body composition and metabolic indicators following a 4-week RCT program in hospitalized obese women with schizophrenia. To our knowledge, this is the first RCT study on obese patients with schizophrenia by CRD. The results demonstrate that CRD could prevent body weight gain, as well as waistline, hipline, MUAC, TST, FM, and FFM. In addition, TC was the only indicator that decreased after CRD intervention among all metabolic indicators compared with control group. The findings were largely consistent with our proposed research hypothesis.

For the primary research question, the most obvious finding to emerge from the analysis is that all the indicators of body mass and composition of the intervention group decreased. Weight loss of 5% with respect to baseline is generally accepted as a “clinically meaningful” amount (36). The 2013 Obesity Guidelines state that a 3% weight loss could lead to glucose and triglyceride improvements and a 5% weight loss could lead to HDL, LDL, and blood pressure improvements, which are clinically meaningful (37). In present study, the rate of weight loss in the intervention group was 2.95%, which did not meet the minimum recommended target. The short-term duration of the intervention was the primary explanation, which is the shortcoming of this study. CRD with relatively high protein contents may facilitate weight loss due to increased satiety and sustained energy expenditure via diet induced thermogenesis (38). We offered adequate protein for the CRD diet, but this did not prevent the loss of FFM. This may be due to the low energy intake of the CRD group, which resulted in gluconeogenesis by breaking down endogenous proteins. In accordance with this result, previous studies with general population also demonstrated the loss of FFM after CRD (31, 39). There is a general agreement that the loss of FFM should be avoided due to FFM’s protective effect against insulin resistance (28). A study in overweight participant showed that CRD encompassing endurance exercises could attenuate FFM loss at a similar degree of weight loss (40).

In addition, the present finding that BW, BMI, WC, HC, TST, and MUAC in the control group increased slightly at the end of the experiment compared with the baseline. This result indicates that there is excess energy intake in hospitalized patients with schizophrenia, which is mainly attributable to increased appetite or behavior inhibition caused by antipsychotics (41). It is commonly recognized that maintaining the weight loss is challenging for a person who returns to the same environment and behaviors that produced their weight gain (42). Undoubtedly, for obese persons with schizophrenia avoiding weight regain requires overcoming additional obstacles.

Besides changes of body weight and composition, CRD also contributed to commensurate changes in other metabolic indicators. For example, Hietaniemi et al. observed after 8-week CRD on women with obesity the triglyceride and fasting insulin concentrations decreased significantly (43). Rothberg et al. reported the improvements in blood lipid profile and the lowering of BP in those patients who had the WC decreasing after low-calorie diet (LCD) intervention. Rothberg et al. reported improved lipid profiles and decreased blood pressure in subjects who had declined WC after the LCD intervention (44). In current study, only TC observed a significant difference between intervention group and control group. Indicators other than TG and HDL-C, although there are statistical differences in the comparison before and after the experiment within the group, no difference in changes between the groups was found. Four weeks of intervention may not be sufficient to cause changes in metabolic outcomes. It has been shown that metabolic changes can only be produced when the rate of weight loss exceeds 5% or more (45), while the rate of weight loss in this study was only 2.95%. Furthermore, the blood glucose, lipid profiles, and blood pressure of the subjects were at the normal level when they were enrolled, resulting in their insensitivity to dietary intervention (46, 47). One unanticipated finding was that FBG decreased both in intervention group and control group, which were medium and small, respectively. The provision of health education to all participants could be the reason why both groups decreased; other reasons could be type 1 error of hypothesis testing.

To eliminate the possible influence of the wide age range of the study population on the results. We performed subgroup analyses for different age groups. We found that TC changes in the younger subgroup only differed more between the two intervention groups. There were also differences in blood pressure between two intervention methods in the middle-aged group. This suggests that age factors influence the effect of CRD intervention on metabolic status. Due to the limited sample size of each subgroup, the findings of the subgroup analysis need further validation.

This study has certain limitations and deficiencies, and the results of the study should be interpreted carefully. First, given the excess of female patients with schizophrenia over males and the stronger willingness of females to lose weight, we selected only female patients as the study population. Moreover, the study was conducted in a closed hospital setting, which can ensure the compliance of subjects, resulting in limited extrapolation of the findings. Secondly, this study did not carry out exercise intervention for the study subjects, obesity intervention should be carried out in many ways, especially diet and exercise. In addition, the duration of the intervention in this study was short, due to the average length of stay of the patients being 50 days, and the intervention subjects were not followed up, so the long-term effects of the intervention lacked evaluation. Weight regains after returning to normal or habitual diets is a common problem affecting the long-term effectiveness of dietary interventions.

## 5. Conclusion

In conclusion, we provide evidence that calorie-restricted diets improve weight and metabolic markers in obese patients hospitalized with schizophrenia in women. Future studies should include larger trials with long-term follow-up and a focus on weight maintenance following initial intervention.

## Data availability statement

The raw data supporting the conclusions of this article will be made available by the authors, without undue reservation.

## Ethics statement

The studies involving human participants were reviewed and approved by the human ethics committees of the Second Affiliated Hospital of Xinxiang Medical University. The patients/participants provided their written informed consent to participate in this study.

## Author contributions

LZ and YH: conceptualization. LZ and CY: methodology. LZ, MZ, ZZ, and XL: investigation. YH and LZ: writing—original draft preparation. CY and PH: validation. LL and PH: writing—review and editing. YH and CY: supervision. All authors read and agreed to the published version of the manuscript.

## Funding

This research was funded by the National Natural Science Foundation of China (81703216), the Open Project of Henan Key Lab of Biological Psychiatry (ZDSYS2021005), and the Joint Co-construction Project of Henan Medical Science and Technology Research Plan (LHGJ20220637).

## Conflict of interest

The authors declare that the research was conducted in the absence of any commercial or financial relationships that could be construed as a potential conflict of interest.

## Publisher’s note

All claims expressed in this article are solely those of the authors and do not necessarily represent those of their affiliated organizations, or those of the publisher, the editors and the reviewers. Any product that may be evaluated in this article, or claim that may be made by its manufacturer, is not guaranteed or endorsed by the publisher.

## Footnotes

^1^ http://www.chictr.org.cn
