# Supplementary material for: Calorie-restricted diet mitigates weight gain and metabolic abnormalities in obese women with schizophrenia: a randomized controlled trial
Source: Front Nutr. 2023 May 5;10:1038070. doi: 10.3389/fnut.2023.1038070 (PMC10198382; doi:10.3389/fnut.2023.1038070)
Supplement: Supplementary file 2 [file Data_Sheet_1.docx]

**Supplementary Table 1.** Seven-day average calorie and macronutrient intakes for the CRD and ND groups (Mean ± SD)

| Dietary intake | CRD | Calorie (%) | ND | Calorie (%) |
| --- | --- | --- | --- | --- |
| Calorie, kcal | 1470.75±130.09 | 100.00% | 1945.11±96.71 | 100.00% |
| Protein, g | 64.76±7.84 | 17.61% | 57.69±7.99 | 11.86% |
| Fat, g | 43.42±4.15 | 26.57% | 52.89±7.96 | 24.47% |
| Carbohydrates, g | 205.23±22.09 | 55.82% | 309.59±27.55 | 63.67% |
| Cholesterol, mg | 320.02±50.75 | - | 280.45±58.09 | - |

Abbreviation: CRD, calorie-restricted diet; ND, normal diet.

**Supplementary Table 2.** Changes in body weight and composition during the study in the CRD intervention group (n=15) vs. the ND group (n=20) in subset of 20 ≤ age < 30 (mean ± SD)

| Variables | Group | Baseline | End-of-trial | Change | *p*^b^ | Cohen’*d* | *p*^b^ | *ηp*^2^ |
| --- | --- | --- | --- | --- | --- | --- | --- | --- |
| BW, kg | ND | 81.70 ± 8.49 | 82.61 ± 8.34 | -0.91 ± 1.98 | 0.055 | 0.108 | <0.001 | 0.628 |
|  | CRD | 84.62 ± 11.52 | 81.32 ± 11.18 | 3.30 ± 0.97 | <0.001 | 0.291 |  |  |
| BMI, kg/m^2^ | ND | 31.18 ± 2.18 | 31.55 ± 2.47 | -0.37 ± 0.81 | 0.055 | 0.158 | <0.001 | 0.589 |
|  | CRD | 33.12 ± 3.74 | 31.82 ± 3.57 | 1.30 ± 0.40 | <0.001 | 0.355 |  |  |
| WC, cm | ND | 108.08 ± 7.03 | 109.31 ± 6.91 | -1.23 ± 2.13 | 0.018 | 0.176 | <0.001 | 0.745 |
|  | CRD | 108.31 ± 8.04 | 102.46 ± 6.19 | 5.85 ± 2.77 | <0.001 | 0.816 |  |  |
| HC, cm | ND | 110.89 ± 6.91 | 111.87 ± 7.06 | -0.99 ± 1.56 | 0.011 | 0.141 | <0.001 | 0.626 |
|  | CRD | 112.30 ± 7.77 | 108.62 ± 7.33 | 3.68 ± 2.15 | <0.001 | 0.487 |  |  |
| MUAC, cm | ND | 31.79 ± 3.27 | 32.37 ± 3.38 | -0.58 ± 0.87 | 0.008 | 0.173 | <0.001 | 0.416 |
|  | CRD | 32.37 ± 2.90 | 30.66 ± 3.78 | 1.71 ± 1.90 | 0.004 | 0.508 |  |  |
| TST, cm | ND | 31.97 ± 5.64 | 32.95 ± 5.38 | -0.99 ± 1.19 | 0.002 | 0.179 | <0.001 | 0.561 |
|  | CRD | 34.42 ± 7.60 | 29.14 ± 6.62 | 5.28 ± 4.13 | <0.001 | 0.741 |  |  |
| FM, kg | ND | 31.14 ± 4.66 | 31.59 ± 4.39 | -0.45 ± 1.37 | 0.158 | 0.099 | <0.001 | 0.491 |
|  | CRD | 32.75 ± 6.05 | 30.43 ± 5.11 | 2.32 ± 1.68 | <0.001 | 0.414 |  |  |
| FFM, kg | ND | 45.98 ± 3.90 | 46.44 ± 4.04 | -0.46 ± 0.89 | 0.033 | 0.116 | 0.004 | 0.234 |
|  | CRD | 47.05 ± 5.66 | 46.17 ± 5.76 | 0.88 ± 1.56 | 0.046 | 0.154 |  |  |

Abbreviation: *ηp*^2^, partial eta squared effect size, BW, body weight; BMI, body mass index; WC, waist circumference; HC, hip circumference; MUAC, mid-upper arm circumference; TST, triceps skinfold thickness; FM, fat mass; FFM, free-fat mass.

**Supplementary Table 3.** Changes in body weight and composition during the study in the CRD intervention group (n=17) vs. the ND group (n=14) in subset of 30 ≤ age < 45 (mean ± SD)

| Variables | Group | Baseline | End-of-trial | Change | *p*^b^ | Cohen’*d* | *p*^b^ | *ηp*^2^ |
| --- | --- | --- | --- | --- | --- | --- | --- | --- |
| BW, kg | ND | 78.26 ± 6.69 | 78.15 ± 7.04 | 0.11 ± 2.05 | 0.838 | 0.017 | 0.004 | 0.260 |
|  | CRD | 77.59 ± 5.96 | 75.58 ± 5.99 | 2.01 ± 1.21 | <0.001 | 0.336 |  |  |
| BMI, kg/m^2^ | ND | 30.86 ± 2.11 | 30.80 ± 2.09 | 0.06 ± 0.85 | 0.797 | 0.028 | 0.004 | 0.261 |
|  | CRD | 30.80 ± 1.95 | 29.99 ± 1.82 | 0.81 ± 0.51 | <0.001 | 0.428 |  |  |
| WC, cm | ND | 104.44 ± 6.09 | 104.83 ± 4.95 | -0.39 ± 2.77 | 0.605 | 0.071 | <0.001 | 0.502 |
|  | CRD | 103.26 ± 4.89 | 99.57 ± 5.04 | 3.69 ± 2.03 | <0.001 | 0.744 |  |  |
| HC, cm | ND | 107.11 ± 4.29 | 107.31 ± 3.49 | -0.20 ± 2.22 | 0.741 | 0.051 | <0.001 | 0.384 |
|  | CRD | 106.76 ± 4.12 | 104.07 ± 4.28 | 2.69 ± 1.92 | <0.001 | 0.640 |  |  |
| MUAC, cm | ND | 31.91 ± 1.86 | 31.22 ± 2.00 | 0.69 ± 0.84 | 0.009 | 0.355 | 0.210 | 0.056 |
|  | CRD | 32.34 ± 2.18 | 30.87 ± 1.66 | 1.47 ± 1.90 | 0.006 | 0.759 |  |  |
| TST, cm | ND | 30.39 ± 5.06 | 31.52 ± 3.96 | -1.13 ± 3.39 | 0.235 | 0.248 | <0.001 | 0.408 |
|  | CRD | 30.45 ± 4.64 | 27.79 ± 4.17 | 2.66 ± 2.14 | <0.001 | 0.603 |  |  |
| FM, kg | ND | 28.49 ± 3.91 | 28.58 ± 3.81 | -0.09 ± 1.79 | 0.849 | 0.024 | 0.044 | 0.137 |
|  | CRD | 29.31 ± 3.23 | 28.07 ± 2.95 | 1.24 ± 1.51 | 0.004 | 0.402 |  |  |
| FFM, kg | ND | 45.06 ± 3.77 | 44.96 ± 3.81 | 0.11 ± 1.74 | 0.821 | 0.028 | 0.245 | 0.048 |
|  | CRD | 78.26 ± 6.69 | 78.15 ± 7.04 | 0.58 ± 0.95 | 0.024 | 0.175 |  |  |

Abbreviation: *ηp*^2^, partial eta squared effect size, WB, body weight; BMI, body mass index; WC, waist circumference; HC, hip circumference; MUAC, mid-upper arm circumference; TST, triceps skinfold thickness; FM, fat mass; FFM, free-fat mass.

**Supplementary Table 4.** Changes in body weight and composition during the study in the CRD intervention group (n=13) vs. the ND group (n=11) in subset of 45 ≤ age ≤ 60 (mean ± SD)

| Variables | Group | Baseline | End-of-trial | Change | *p*^b^ | Cohen’*d* | *p*^b^ | *ηp*^2^ |
| --- | --- | --- | --- | --- | --- | --- | --- | --- |
| BW, kg | ND | 76.05 ± 3.26 | 77.86 ± 4.18 | -1.82 ± 2.20 | 0.021 | 0.485 | <0.001 | 0.495 |
|  | CRD | 79.99 ± 7.70 | 78.20 ± 7.35 | 1.79 ± 1.23 | <0.001 | 0.238 |  |  |
| BMI, kg/m^2^ | ND | 30.67 ± 0.99 | 31.39 ± 1.09 | -0.72 ± 0.88 | 0.021 | 0.692 | <0.001 | 0.490 |
|  | CRD | 31.74 ± 2.04 | 31.04 ± 1.99 | 0.71 ± 0.47 | <0.001 | 0.350 |  |  |
| WC, cm | ND | 104.64 ± 3.18 | 106.56 ± 3.23 | -1.93 ± 2.33 | 0.021 | 0.602 | 0.001 | 0.431 |
|  | CRD | 108.28 ± 5.07 | 104.84 ± 5.31 | 3.45 ± 3.00 | 0.001 | 0.664 |  |  |
| HC, cm | ND | 106.15 ± 4.81 | 108.23 ± 3.05 | -2.08 ± 2.92 | 0.040 | 0.517 | 0.003 | 0.345 |
|  | CRD | 111.80 ± 4.53 | 107.88 ± 4.92 | 3.92 ± 2.99 | <0.001 | 0.828 |  |  |
| MUAC, cm | ND | 31.74 ± 1.59 | 32.21 ± 1.67 | -0.47 ± 0.82 | 0.084 | 0.290 | 0.019 | 0.234 |
|  | CRD | 32.42 ± 1.76 | 31.97 ± 1.92 | 0.45 ± 0.82 | 0.075 | 0.242 |  |  |
| TST, cm | ND | 27.73 ± 6.23 | 29.42 ± 6.54 | -1.69 ± 2.20 | 0.029 | 0.265 | <0.001 | 0.525 |
|  | CRD | 31.58 ± 5.56 | 28.02 ± 5.25 | 3.57 ± 2.49 | <0.001 | 0.660 |  |  |
| FM, kg | ND | 29.15 ± 2.10 | 30.12 ± 2.11 | -0.97 ± 1.25 | 0.027 | 0.463 | 0.005 | 0.320 |
|  | CRD | 31.55 ± 3.64 | 30.34 ± 3.52 | 1.22 ± 1.47 | 0.012 | 0.339 |  |  |
| FFM, kg | ND | 42.62 ± 1.73 | 43.39 ± 2.62 | -0.77 ± 1.28 | 0.073 | 0.348 | 0.014 | 0.257 |
|  | CRD | 44.09 ± 4.25 | 43.62 ± 4.20 | 0.48 ± 0.92 | 0.087 | 0.113 |  |  |

Abbreviation: *ηp*^2^, partial eta squared effect size, WB, body weight; BMI, body mass index; WC, waist circumference; HC, hip circumference; MUAC, mid-upper arm circumference; TST, triceps skinfold thickness; FM, fat mass; FFM, free-fat mass.

**Supplementary Table 5.** Changes in metabolic markers during the study in the CRD intervention group (n=15) vs. the ND group (n=20) in subset of 20 ≤ age < 30 (mean ± SD)

| Variables | Group | Baseline | End-of-trial | Change | *p*^b^ | Cohen’d | *p*^b^ | *ηp*^2^ |
| --- | --- | --- | --- | --- | --- | --- | --- | --- |
| FBG, mmol/L | ND | 4.94 ± 0.67 | 4.73 ± 0.78 | 0.21 ± 0.94 | 0.322 | 0.292 | 0.221 | 0.046 |
|  | CRD | 4.95 ± 0.72 | 4.45 ± 0.54 | 0.50 ± 0.54 | 0.003 | 0.786 |  |  |
| TC, mmol/L | ND | 4.55 ± 0.75 | 4.24 ± 0.85 | 0.31 ± 0.83 | 0.113 | 0.382 | 0.022 | 0.152 |
|  | CRD | 4.82 ± 0.78 | 3.83 ± 0.54 | 0.99 ± 0.65 | <0.001 | 1.483 |  |  |
| TG, mmol/L | ND | 1.62 ± 0.66 | 2.07 ± 1.01 | -0.45 ± 1.09 | 0.079 | 0.529 | 0.094 | 0.085 |
|  | CRD | 2.65 ± 2.80 | 1.71 ± 0.66 | 0.94 ± 2.46 | 0.161 | 0.463 |  |  |
| HDL-C, mmol/L | ND | 1.14 ± 0.20 | 1.18 ± 0.35 | -0.04 ± 0.32 | 0.535 | 0.156 | 0.079 | 0.093 |
|  | CRD | 1.18 ± 0.37 | 1.02 ± 0.35 | 0.16 ± 0.32 | 0.070 | 0.454 |  |  |
| LDL-C, mmol/L | ND | 2.75 ± 0.61 | 2.49 ± 0.63 | 0.26 ± 0.58 | 0.063 | 0.414 | 0.343 | 0.028 |
|  | CRD | 2.76 ± 0.85 | 2.34 ± 0.52 | 0.42 ± 0.63 | 0.021 | 0.597 |  |  |
| SBP, mmHg | ND | 114.40 ± 11.02 | 114.20 ± 10.73 | 0.20 ± 12.25 | 0.943 | 0.018 | 0.086 | 0.089 |
|  | CRD | 117.67 ± 14.07 | 109.73 ± 10.79 | 7.93 ± 11.31 | 0.017 | 0.633 |  |  |
| DBP, mmHg | ND | 74.80 ± 6.53 | 74.95 ± 7.09 | -0.15 ± 9.20 | 0.943 | 0.022 | 0.690 | 0.005 |
|  | CRD | 75.67 ± 7.92 | 74.13 ± 6.97 | 1.53 ± 8.93 | 0.517 | 0.206 |  |  |

Abbreviation: FBG, fasting blood-glucose; TC, total cholesterol; TG, triglycerides; HDL-C, high density lipoprotein-cholesterol; LDL-C, low density lipoprotein-cholesterol; SBP, systolic blood pressure; DBP, diastolic blood pressure.

**Supplementary Table 6.** Changes in metabolic markers during the study in the CRD intervention group (n=17) vs. the ND group (n=14) in subset of 30 ≤ age < 45 (mean ± SD)

| Variables | Group | Baseline | End-of-trial | Change | *p*^b^ | Cohen’d | *p*^b^ | *ηp*^2^ |
| --- | --- | --- | --- | --- | --- | --- | --- | --- |
| FBG, mmol/L | ND | 5.20 ± 0.68 | 4.75 ± 0.87 | 0.45 ± 0.59 | 0.015 | 0.572 | 0.676 | 0.006 |
|  | CRD | 5.21 ± 0.78 | 4.84 ± 0.66 | 0.36 ± 0.56 | 0.017 | 0.502 |  |  |
| TC, mmol/L | ND | 4.67 ± 1.37 | 4.38 ± 0.83 | 0.29 ± 1.27 | 0.407 | 0.255 | 0.271 | 0.043 |
|  | CRD | 5.02 ± 0.57 | 4.20 ± 0.72 | 0.82 ± 0.65 | <0.001 | 1.259 |  |  |
| TG, mmol/L | ND | 1.45 ± 0.65 | 1.86 ± 0.79 | -0.41 ± 0.67 | 0.039 | 0.571 | 0.711 | 0.005 |
|  | CRD | 2.04 ± 1.60 | 2.08 ± 0.88 | -0.04 ± 1.66 | 0.921 | 0.031 |  |  |
| HDL-C, mmol/L | ND | 1.18 ± 0.21 | 1.12 ± 0.24 | 0.06 ± 0.19 | 0.274 | 0.264 | 0.595 | 0.010 |
|  | CRD | 1.14 ± 0.22 | 1.06 ± 0.24 | 0.08 ± 0.28 | 0.263 | 0.346 |  |  |
| LDL-C, mmol/L | ND | 2.61 ± 0.66 | 2.59 ± 0.62 | 0.02 ± 0.55 | 0.886 | 0.033 | 0.108 | 0.090 |
|  | CRD | 3.07 ± 0.66 | 2.49 ± 0.55 | 0.58 ± 0.64 | 0.002 | 0.946 |  |  |
| SBP, mmHg | ND | 119.71 ± 11.03 | 123.86 ± 11.32 | -4.14 ± 17.02 | 0.379 | 0.371 | 0.011 | 0.210 |
|  | CRD | 121.53 ± 11.44 | 114.06 ± 8.40 | 7.47 ± 12.43 | 0.025 | 0.744 |  |  |
| DBP, mmHg | ND | 77.71 ± 6.34 | 78.86 ± 9.57 | -1.14 ± 10.41 | 0.688 | 0.141 | 0.014 | 0.196 |
|  | CRD | 80.71 ± 7.66 | 72.41 ± 7.06 | 8.29 ± 7.38 | <0.001 | 1.126 |  |  |

Abbreviation: FBG, fasting blood-glucose; TC, total cholesterol; TG, triglycerides; HDL-C, high density lipoprotein-cholesterol; LDL-C, low density lipoprotein-cholesterol; SBP, systolic blood pressure; DBP, diastolic blood pressure.

**Supplementary Table 7.** Changes in metabolic markers during the study in the CRD intervention group (n=13) vs. the ND group (n=11) in subset of 45 ≤ age ≤ 60 (mean ± SD)

| Variables | Group | Baseline | End-of-trial | Change | *p*^b^ | Cohen’d | *p*^b^ | *ηp*^2^ |
| --- | --- | --- | --- | --- | --- | --- | --- | --- |
| FBG, mmol/L | ND | 5.03 ± 0.64 | 4.86 ± 0.65 | 0.17 ± 0.66 | 0.425 | 0.255 | 0.902 | 0.001 |
|  | CRD | 5.97 ± 0.90 | 5.06 ± 0.64 | 0.91 ± 1.08 | 0.010 | 1.168 |  |  |
| TC, mmol/L | ND | 4.78 ± 0.77 | 4.87 ± 1.14 | -0.09 ± 1.19 | 0.803 | 0.094 | 0.381 | 0.037 |
|  | CRD | 4.72 ± 0.75 | 4.53 ± 0.66 | 0.19 ± 0.58 | 0.267 | 0.265 |  |  |
| TG, mmol/L | ND | 1.73 ± 0.66 | 2.29 ± 1.32 | -0.56 ± 0.97 | 0.082 | 0.541 | 0.212 | 0.073 |
|  | CRD | 1.46 ± 0.89 | 1.64 ± 0.70 | -0.18 ± 0.53 | 0.241 | 0.228 |  |  |
| HDL-C, mmol/L | ND | 1.32 ± 0.26 | 1.19 ± 0.23 | 0.13 ± 0.30 | 0.178 | 0.524 | 0.486 | 0.023 |
|  | CRD | 1.15 ± 0.20 | 1.12 ± 0.25 | 0.03 ± 0.37 | 0.763 | 0.139 |  |  |
| LDL-C, mmol/L | ND | 2.69 ± 0.59 | 2.86 ± 0.85 | -0.17 ± 0.91 | 0.549 | 0.233 | 0.503 | 0.022 |
|  | CRD | 3.01 ± 0.70 | 2.87 ± 0.63 | 0.14 ± 0.38 | 0.212 | 0.210 |  |  |
| SBP, mmHg | ND | 118.91 ± 12.13 | 114.18 ± 13.87 | 4.73 ± 12.31 | 0.231 | 0.363 | 0.286 | 0.054 |
|  | CRD | 120.15 ± 14.09 | 119.31 ± 8.73 | 0.85 ± 13.31 | 0.823 | 0.072 |  |  |
| DBP, mmHg | ND | 76.09 ± 9.99 | 74.73 ± 7.98 | 1.36 ± 12.10 | 0.716 | 0.151 | 0.293 | 0.052 |
|  | CRD | 77.85 ± 9.08 | 78.54 ± 7.62 | -0.69 ± 6.34 | 0.701 | 0.083 |  |  |

Abbreviation: FBG, fasting blood-glucose; TC, total cholesterol; TG, triglycerides; HDL-C, high density lipoprotein-cholesterol; LDL-C, low density lipoprotein-cholesterol; SBP, systolic blood pressure; DBP, diastolic blood pressure.
